# INCF plasmids responsible by dissemination of *bla*_KPC_ gene among enterobacteriaceae

**DOI:** 10.1186/1753-6561-5-S6-P132

**Published:** 2011-06-29

**Authors:** F Calisto, L Lito, J Melo-Cristino, A Duarte

**Affiliations:** 1 i-Med, Faculdade de Farmàcia, CHLN, EPE Lisboa, Lisbon, Portugal; 2 Laboratory of Microbiology, CHLN, EPE Lisboa, Lisbon, Portugal

## Introduction / objectives

In recent years, carbapenem resistance has emerged among *Enterobacteriaceae* in many geographical locations due to *Klebsiella pneumoniae* producing KPC carbapenemase. The aim of this study was to characterize the genetic elements involved in *bla*_KPC_ gene mobilization and diffusion.

## Methods

A total of 22 isolates, *K. pneumoniae* (n=20), *Escherichia coli* (n=1) and *Enterobacter aerogenes* (n=1), were collected from a university hospital, in Lisbon, Portugal. MIC were determined by Etest and interpreted according to Clinical and Laboratory Standards Institute guidelines. PCR was performed with primers designed to amplify *bla*_SHV_, *bla*_TEM_ and *bla*_KPC_ genes. Molecular typing was performed by M13-PCR fingerprinting and in representative strains by Multilocus Sequence Typing (MLST). Replicon typing was used to define plasmid incompatibility groups (Inc).

## Results

Twenty-two isolates had MIC ranging between 2 and 32 mg/L for imipenem and meropenem. All isolates encoding KPC-3. Associated to carbapenemase 7 isolates encoded for TEM-1 or SHV-1 and 6 isolates for both TEM-1 and SHV-type. Although M13-PCR fingerprint analysis showed four predominant profiles, but with MLST only one sequence type ST11 was found among *K. pneumoniae* isolates. The predominant plasmid was included in IncF (90.5%) and was found plasmids belong to different replicons FIC (52.4%), HI1, HI2, and I1-Iγ (47.8%), T (42.8%), FIIAs (33.3%), W and P (32.8%) and FIB (9.5%).

## Conclusion

The *bla*_KPC_ gene, often associated with other β-lactamases, was transferred between *K. pneumoniae*, *E. aerogenes* and *E. coli* strains by IncF group plasmid. The clustering of resistance genes in plasmids and their organization with regard to cotransfer underlines the importance of these plasmids in the spread of antimicrobial multiresistance.

## Disclosure of interest

None declared.

